# Federated Transfer Learning for Authentication and Privacy Preservation Using Novel Supportive Twin Delayed DDPG (S-TD3) Algorithm for IIoT

**DOI:** 10.3390/s21237793

**Published:** 2021-11-23

**Authors:** Arumugam K, Srimathi J, Sudhanshu Maurya, Senoj Joseph, Anju Asokan, Poongodi M, Abdullah A. Algethami, Mounir Hamdi, Hafiz Tayyab Rauf

**Affiliations:** 1 Department of Computer Science, Karpagam Academy of Higher Education, Coimbatore 641021, India; aaruk.dpm@gmail.com; 2 Vivekanandha Institute of Information and Management Studies, Elaiyampalayam 637205, India; sriphd2020@gmail.com; 3 School of Computing, Bhimtal Campus, Graphic Era Hill University, Uttarkhand 248001, India; dr.sm0302@gmail.com; 4 Department of Electronics and Communication Engineering, Sri Krishna College of Technology, Kovaipudar, Coimbatore 641042, India; senojjoseph@skct.edu.in (S.J.); anjuasokan@skct.edu.in (A.A.); 5 Division of Information and Computing Technology, College of Science and Engineering, Hamad Bin Khalifa University, Qatar Foundation, Doha 582500, Qatar; mhamdi@hbku.edu.qa; 6 Department of Engineering, Taif University, Taif 26312, Saudi Arabia; a_algethami@tu.edu.sa; 7 Department of Computer Science, Faculty of Engineering, University of Bradford, Bradford BD7-01274, UK; h.rauf4@bradford.ac.uk

**Keywords:** Internet of Things, authentication, privacy, security services

## Abstract

The Industrial Internet of Things (IIoT) has led to the growth and expansion of various new opportunities in the new Industrial Transformation. There have been notable challenges regarding the security of data and challenges related to privacy when collecting real-time and automatic data while observing applications in the industry. This paper proposes an Federated Transfer Learning for Authentication and Privacy Preservation Using Novel Supportive Twin Delayed DDPG (S-TD3) Algorithm for IIoT. In FT-Block (Federated transfer learning blockchain), several blockchains are applied to preserve privacy and security for all types of industrial applications. Additionally, by introducing the authentication mechanism based on transfer learning, blockchains can enhance the preservation and security standards for industrial applications. Specifically, Novel Supportive Twin Delayed DDPG trains the user model to authenticate specific regions. As it is considered one of the most open and scalable interacting platforms of information, it successfully helps in the positive transfer of different kinds of data between devices in more significant and local operations of the industry. It is mainly due to a single authentication factor, and the poor adaptation to regular increases in the number of users and different requirements that make the current authentication mechanism suffer a lot in IIoT. As a result, it has been very clearly observed that the given solutions are very useful.

## 1. Introduction

**Figure 1:**
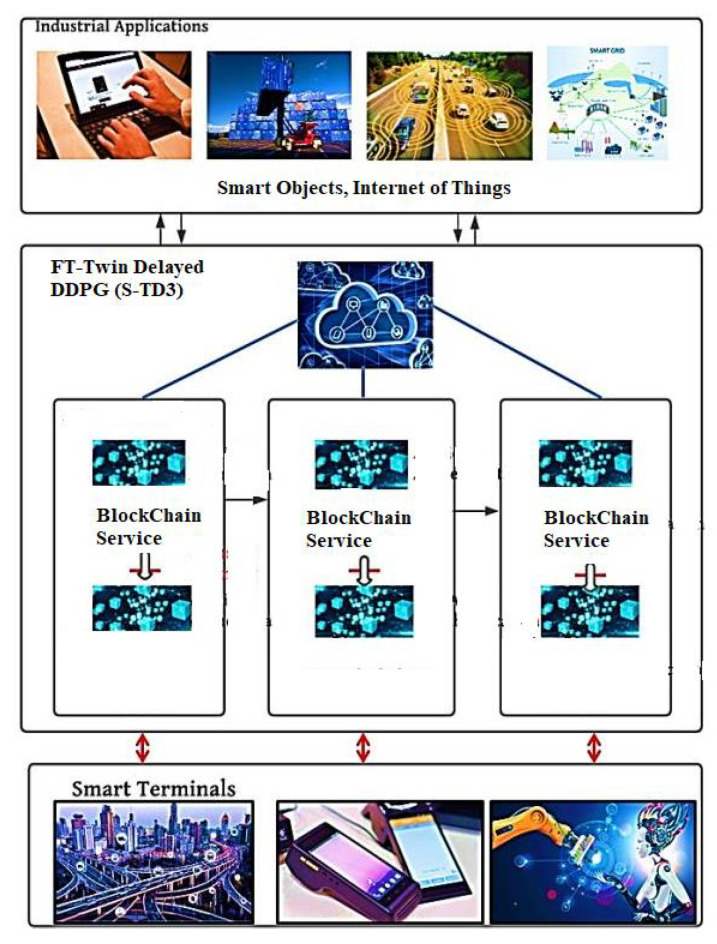
The framework of FT-Twin Delayed DDPG(S-TD3).

Nowadays, essential applications of IoT have created great opportunities for industrial innovations [1]. It integrates mobile communications, cloud computing, and artificial intelligence into all elements of the production process. IIoT successfully helps in the positive transfer of different kinds of data between devices in industries within larger and local operations of the industry [2,3]. The huge number of data generated by the devices connected to it has brought forward their needs for more efficient and accurate automatic and real-time collection, examination, and processing of data. A lot of attention and focus have been given towards all of the challenges faced in relation to the security of data and to privacy [4]. To successfully overcome every challenge in this field, a large number of research was conducted in the industry and proper development processes are adopted across the world, such as intelligence edge [5], transfer learning [6], and blockchain [7]. As as innovative and technological paradigm has been created, artificial intelligence (AI) and its edge help integrate mobile computing, AI, and edge caching in relation to the number of end users [8]. In IIoT that is enabled by edge intelligence, the management of edge resources is controlled by AI. It can lead to strong computation and can increase the storage of data in edge networks. It also meets the constraints for delays and all other types of requirements for all of the performances that are useful in industrial applications. The requirements for evaluations of large sets of data and more investments towards computation have decreased due to its applications in the developmental process of the model. Transfer learning helps a lot in exploring and conducting experiments that help develop new and innovating ideas and help increase productivity. In IIoT, combining transfer learning and block chain helps boost the performances made by both transfer learning and block chains. With the help of blockchains, the credibility of data sources can be easily verified. As a result, it helps in preventing data from being tampered and stops fraudulent actions from being carried out using the data. Intelligent terminal applications in the industry are considered fully responsible for collecting efficient and complete data of the industry. Transfer learning is a more efficient methodology over a deep learning network on small data sets. Anomaly-based detection with deep learning is a recent approach proposed by [9]. Enriching transfer learning with neural translation deployment was introduced in [10], where the features of a lexicon were introduced with transfer learning with a shared vocabulary. The author in [11] proposed the semi-supervised attention model, which helps in discriminating feature embedding, and a high accuracy rate was achieved. The enhanced authentication protocol was introduced in that research [12], and a new method of authentication and key exchange protocol was discovered to give an efficient authentication mechanism for IoT. Several hurdles have acted as barriers to the creation of blockchains and TL in the technology of IIoT. For example, in the old process of blockchains, users can use a single process of authentication to pass the process. This means that malicious actors can pass the authentication process if they passed them in a specific region. Based on the above analysis, the IIoT architecture is constructed via empowerment by edge and via intelligence and employment of the authentication process based on TL. This is performed in order to build trustworthy blockchains and intelligence. It is shown in Figure 1 that the proposed architecture possesses three or four aspects: intelligent terminals, industrial applications, IIoT applications and edge, and intelligent networks. All of the intelligent terminals of industrial applications are responsible for collecting industrial data that is reliable and efficient. The edge server is a component of the edge intelligent network, blockchain system, and artificial intelligence [11]. They are responsible for analysis, fusion, and processing of the data; for providing security to data; and for protecting its privacy for many IIoT applications. An authentication method with Novel Supportive Twin Delayed DDPG has been developed in accordance with the planned IIoT architecture with a formulated novel mechanism for FT-Block.

All of the essential contributions that have been conducted with the help of this paper are highlighted below:

- The privacy of a user’s various blockchains that have been brought forward to authenticate the mechanism of the users is successfully and efficiently preserved so that it protects them from all attacks [11]. The blockchains that introduced are the inner as well as the outer blockchains.
- The implementation of the authentication of the users in every part is completely dependent on the credit of the users. All this is carried out to gain more and more accuracy towards the process of authentication. It is the authenticated mechanism design that helps in merging the credit of the users with both the local credit as well as the crossed region credits. Some algorithms are created and used to bring more and more accuracy by providing proper training to the local models of authentication. A Supportive Twin Delayed DDPG (S-TD3) algorithm is then created for training the local model of authentication with utmost accuracy.
- To decrease the extra time that is spent in the models of authentication, the method of transfer learnings (TL) is applied. With the help of transfer learning of the outer blockchains, the successful transfer of the models of authentication is accurately carried out from the local level to foreign users.
- The final results of these are (i) that there is accurate authentication for local as well as foreign users, and (ii) that higher throughput and low latency are achieved in several scenarios of IIoT.

## 2. Related Work

At present, a blockchain facilitated machine learning authentication programme has gained much importance in the Internet of Things. In [13], authentication has been proposed based on mobile edge computing according to the technology of blockchain. In this, one-way hash functions, and rotation bitwise and XOR operations take place. A smart contract was designed by adopting machine learning and blockchain as it can remove data trading for third parties [14,15]. It authenticates and authorizes the owner and purchaser of data through the download mechanism known as off-chain. In [16,17], the problems involved in centralized record keeping and decentralized case computation were considered. After that, a mechanism for authentication helped improve the healthcare record-keeping by enhancing reliability. A multiple-wireless sensor network-based authentication was established on the blockchain [18,19]. Several inner and outer blockchains were introduced in this research work that help integrate the local as well as public chain and form a hybrid blockchain design. Various types of authentication scenarios were implemented for the formation of the local and public blockchain. In [20,21], edge-based computing was applied to authenticate the design of the system based on improving efficiency and authentication. In the entire system, the blockchain uses a consensus that is tolerant of any faults and helps in designing an authenticating mechanism and tracing the end activity. In [22,23], the author proposed an authenticated system that was assisted by the blockchain for the purpose of preserving privacy in VANETs. The misbehaving vehicles were traced conditionally with a Hyperledger platform that was fabric-based as it enhanced the security and performance criteria. In [24], mechanism for authentication has been proposed for several kinds of fog services as it facilitates data centre authentication. It facilitates light weightiness communication authentication and vehicle animosity and resists any attack on the data centre. In [25,26], proposed a mechanism for authentication based on edge computing-based smart grid blockchains. This mechanism provides reasonable assistance and security. Additionally, TL can reduce the time for model training involved in authentication. In [27,28], proposed an authenticating mechanism that protects against any outside and unauthorized attacks by attackers and facilitates physical authentication. The authentication models facilitate reinforced learning, and TL helps in several discoveries. In [29], a framework for authentication utilizes specific information related to several devices and prevents all types of physical attacks.

## 3. System Model

**Figure 2:**
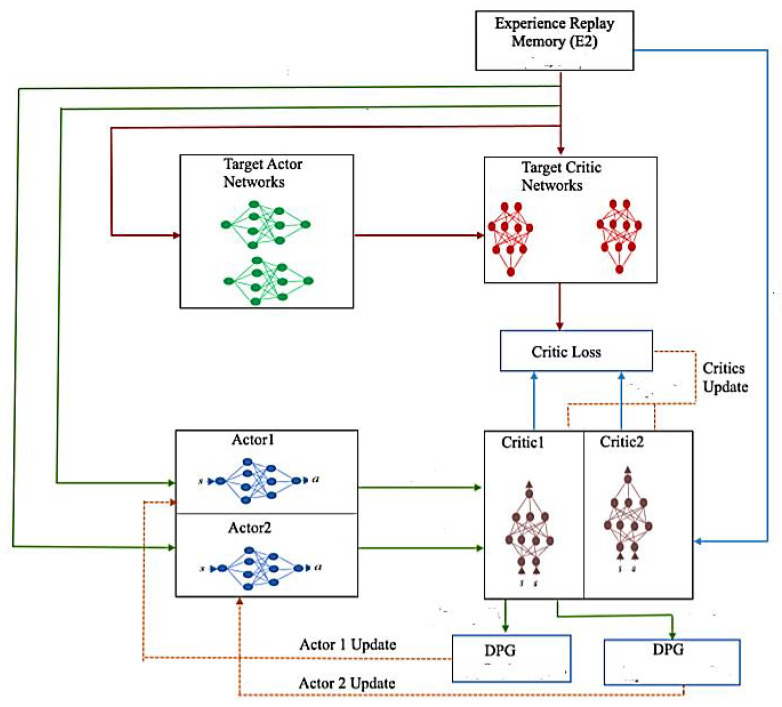
The architecture model of the proposed FT-Block.

In this paper, an efficient mechanism for authentication is formulated that helps in the empowerment of blockchain. It helps construct intelligent and trustworthy blockchains through the creation of multi-tier structures for each of the applications of IoT and creates an internal and outer blockchain structure [11]. Additionally, the blockchain helps in authenticating and enhancing the usability of local users in several regions. In contrast, the outer blockchain authenticates the users that operate from a foreign country. All of the servers for authentication, and the CA(Certificate Authority) of block chains and servers are used on the edge server for the resources that are required for computation. Figure 2 presents the system model and states that there are several components:

The users plays a significant role in connecting with the innermost or outermost blockchain if they successfully clear the authentication test [11]. *A* user who works efficiently in the $R_{j}$ enhances their innermost credit and helps the user clear their authentication of $R_{i}$. When the user moves from $R_{i}$ to $R_{j}$, they might fail to authenticate in $R_{j}$ as different authentication standards vary from one region to the other.

The two types of CA are discussed here. First one represents an internal blockchain, known as the i_CA, and the other is the outer blockchain known as the o_CA. The i_CA helps in local user authentication, whereas the outer blockchain facilitates user authentication of foreigners. All types of CAs employ servers that are edged and perform authentication based on DRL in the Internet of Things [11]. All of the CAs are useful in withdrawing key pairs of users and helps in eliminating users who use their credit maliciously. There is a problem in the leakage of privacy that has also been considered in this paper.

Moreover, there is a problem in the old blockchain, where users pass the local record for authentication that can act maliciously in other regions. There are two types of attacks that a user may face in the system, and these are as follows:

Attacks are launched by malicious users that help exchange individual tasks [11] for completing and securing sensitive information in the entire blockchain.

Different blockchains can be joined by a malicious user, and they play a role as a legitimate user to obtain information that is sensitive in the entire blockchain.

## 4. The Implementation of the FT-Block

### 4.1. Supportive Twin Delayed DDPG (S-TD3)-Based User Authentication

In the process of user authentication, the credit is chosen as the state. There is a requirement to discover the threshold of credit to determine whether the authentication process must be cleared by the user. The traditional blockchain design passes the user’s authentication; then, the $i_{cr}$ increases locally for good performances and decreases for performances that are not up to par. If the authentication process is not cleared by the user, then the $o_{cr}$ can be improved [11] cross-regionally. Both completion of tasks and reliability of data are essential factors that help in measuring the performance of the users. Then, the $cr_{i}$ of user i can be obtained using Equation (1):

(1) $$Cr_{i}=\gamma \times i\_cr_{i}+\left(1-\gamma \right)\times o\_{cr}_{i}$$

**Figure 3:**
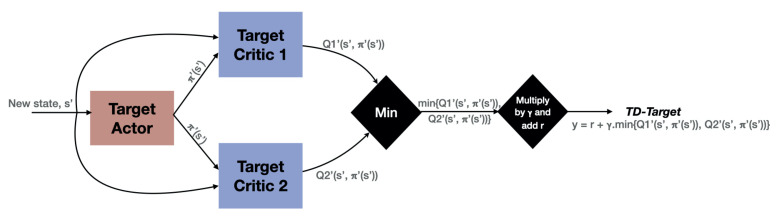
The framework of the proposed Supportive Twin Delayed DDPG (S-TD3).

The system accuracy is improvised using the authentication system developed called Supportive Twin Delayed DDPG Algorithm (S-TD3), which provides higher security for a user based on the authentic mechanism. The proposed method varies from the existing DDPG, which holds *n* number of critic networks $V_{i}s$ and equivalent target critic networks $V_{i}s$, and m number of actor-networks $\varphi_{i}s$ and concerned target actor networks $\varphi_{i}s$, and the attributes are represented by the notations $\eta^{V_{i}}$, $\eta^{{ø}_{i}}$, $\eta^{\varphi_{i}}$, and $\eta^{\theta_{i}}$, correspondingly. The trajectory with the high-return results in the DRL process of learning. Thus, a pool $\upsilon^{*}$, which has the additional experience, is introduced to accumulate the mentioned data of experiences related to traditional ones stockpiled in the pool υ representing the experiences. The Supportive Twin Delayed DDPG (S-TD3) framework is illustrated in Figure 3.

We pre-selected the credit names as the state indicated by *s* during the user authentication process. To specify that the user’s authentication is successful, the score of the credit threshold was discovered within the timeslot *T*. Additionally, the forthcoming state $ST_{t+1}$ was attained by computing the behavior of the user. For instance, the user overcomes the authentication process, the credit i_cr increases at the local level for [11] better performance or goes down otherwise. Additionally, when a user fails to overcome the authentication mechanism, rewards are given if it works well cross-regionally with an improvement in o_cr. Then, the performance of the working model with the *i*th user $P_{i}$ is the profit or is indicated as gain through which they succeeded in the process of authentication. This denotes that the completion of the task represented as (TC) and reliability of data (DR) are primary significant attributes used to determine the performance of user’s model. The user *i*, by assimilating a scale factor β, is given as

(2) $$P_{i}=\beta \times TC_{i}+\left(1-\beta \right)\times DR_{i},$$

where we denote the reward $R_{w}$ as

(3) $$R_{w}=\sum_{i}^{}P_{i}$$

The action $A_{c}$ is calculated according to the supportive network S${}_{1}$( l |$\eta^{S1}$ ), i.e.,

(4) $$A_{c}=(\phi_{1}\left(l_{T}|\eta^{\phi_{1}}\right)+\delta \left(S_{1}\left(l_{T}|\eta^{S_{1}}\right)-\phi_{1}\left(l_{T}|\eta^{\phi_{1}}\right)\right)$$

where 0 ≤δ≤ 1. We use a pool denoted as $\upsilon_{1}$ to accumulate the experiences $l_{t}$, $A_{c}$, $R_{w}$, $l_{t+1}$ and utilize trajectory TR to store $l_{t}$, $A_{c}$.

The model is trained for the proposed network of the Supportive Twin Delayed DDPG (S-TD3) with *M* with the random experience from $\upsilon_{1}^{*}$ as

(5) $$£\left(\eta^{S}\right)=\frac{1}{M}\sum_{i}^{M}|S_{1}\left(l_{i}\right)|\eta^{S_{1}}{)-a_{i}]}^{2}$$

Accordingly, we update the network (critic) with *M* corresponding experiences randomly from

(6) $$£\left(\eta^{S}\right)=\frac{1}{M}\sum_{i}^{M}\left[V_{1}\left(l_{i}\right],a_{i}|\eta^{V_{1}}\right)-y_{i}{]}^{2}$$

where

(7) $$\begin{array}{c} y_{i}=r_{i}+\varepsilon \left[\left(1-\delta \right)S_{1}\left(l_{i+1},\varphi_{i}\left(l_{i+1}|\eta^{\Theta }\right)|\eta^{\emptyset_{1}}\right)\right.\\ +\delta \left(S_{1}\left(l_{i+1},S\left(l_{i+1}|\eta^{\theta_{1}}\right)|\eta^{\emptyset_{1}}\right)\right] \end{array}$$

Then, the gradient policy is implemented to keep track and update the ϕ by

Ultimately, networks (target) $\eta^{\phi^{1}}$ and $\eta^{\theta^{1}}$ efficiently become rationalized with a learning rate denoted with *K*.

It must be noted that the Supportive Twin Delayed DDPG (S-TD3) that is proposed as a learning process is stable and helps in comparing the bootstrapped Deep Deterministic Policy Gradient (DDPG) when doubled [30,31,32,33,34,35]. Initially, the network guides and defines the stability of the supervised Supportive Twin Delayed DDPG (S-TD3) algorithm. The supportive network can be updated by excellent experiences by enhancing the stability of the system. Second, the DDPG is a gradient algorithm that employs the network and uses it as a stabilizer to reduce the oscillations that take place during the entire process of learning. In proposed work, a unsupervised gradient is embraced to enhance the networks that are based on the process towards immediate reward [11]. This is an estimation of the reward function and is an unbiased one. The reward that is received fluctuates immediately and stabilizes in the learning process even if there is an unpredictable environment. Compared with the DDPG, the developed model of Supportive Twin Delayed DDPG (S-TD3) provides an even process of learning that is stable and accelerates the convergence.

### 4.2. Authentication Mechanism on FT-Supervised Supportive Twin Delayed DDPG (S-TD3)

There are 2 types of transfers that are mainly deliberated in this research. The foremost is called the local transfer, which comprises the model, which is helpful in the authentication of local users $R_{i}$ and can be transferred to foreign users and other $R_{i}$. The secon is the cross-region transfer , and it is a model that is trained for the authentication of local users. In general, it can be stated that foreign user authentication [11] helps in determining the users that are qualified for joining the outer blockchain. The authentication process of foreign users aims to define that the users of $R_{j}$, i≠j, are qualified to be connected with the blockchain (outer). Additionally, it is determined that users with the authentication process at the local level of $R_{j}$, all values of *i*, are fit to be connected with the blockchain (inner) [11]. The significance of applying the federated transfer learning process is that it has a high similarity among the authentication processes of users belonging to the local level or to foreign users. Precisely, the resemblance amongst region pairs could be enumerated. For instance, we represent the region *i* $R_{i}$ by a tuple, i.e., $R_{i}$ (task${}_{i}$ , user${}_{i})$, where task${}_{i}$ and user${}_{i}$ denote the set of task and set of user of $R_{i}$. Thus, we could attain the likeness between $R_{i}$ and $R_{j}$ represented by notation Sim${}_{i,j}$ in the formula of distance calculation Mahalanobis, to eliminate the effect of scaling. The sequence of similarity enables the user to define the pair of regions that could be transferred to authentication models in a cross-regional manner [11]. The users that pass authentications related to the specific areas are given credits associated with the related thresholds. As given in Equation (1), it is apparent that the represented weights β must be fixed comparatively >0.5 for users at the local level or <0.5 for users with a foreign authentication. This helps in appreciating both the i_cr as well as the o_cr. It suggests that there is a possibility of transferring the local models in an authenticated manner. In foreign-based user authentication, the overall user performance acts as the state of affairs and serves the entire state and obtains rewarding references [11]. Based on the above similarities, the model for authentication of the user can be transformed to the users of authentication [36,37,38,39,40].

**Figure 4:**
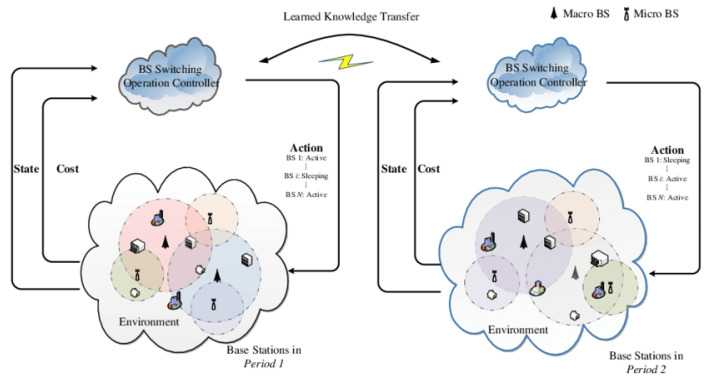
The user authentication: Transfer Learning for Reinforcement learning.

The model [11,12] transferring application is represented in Figure 4. The foremost category of the transfer utilizes the networks by training them for Supportive Twin Delayed DDPG (S-TD3), facilitating user authentication belonging to the category of foreign users. The several attributes of input and layers that are hidden are accomplished with training for enabling the authentication for users belonging to local users and reducing the training time involved in initialization and authentication. However, the parameters of the output layer cannot be shared with the local users, and then, different types of calculations are utilized for performing foreign user authentication. All of the layers can be attuned and transmitted to analyze the results of the authentication. The next transfer is implemented similarly [41,42,43,44,45].

Once this happens at the conclusion of both local and foreign user authentications, all are allowed to access security-level permission. It is possible to provide an effective method for deconstructing and grouping the user integrity of the entire task. Users are classified into various categories according on their security level. After that, all of the sensitive tasks are broken down into several non-sensitive ones and each of them is accompanied by an assured level of security. All of the tasks are accepted only when the level of security is higher for the tasks undertaken. The incentive mechanism can be introduced. Honest users are rewarded with extra points of credit that are related to task-related security levels. The key pairs can be rewarded to CAs and they help in preventing users from stealing information that is sensitive in nature [46,47].

The proposed FT-Block helps in preventing both collusion and Sybil attacks. Once the user has been authenticated, the inner or the outer blockchains can be joined by the users. When the user acts maliciously by pocketing the sensitive information, in turn, their credit drops instantly. When the credit is lower than the threshold, the key pair of the user can be withdrawn, and the lower credit causes the user to have less chances to proceed through authentication. This suggests that the user is not able to cause any other damage to the blockchain by attacking it. Additionally, the authority granted to the user for controlling the outer blockchain is less compared with the inner blockchain. This means that even if two users are from a different blockchain, they often conspire with each other and obtain important data relevant for both the inner and the outer blockchains.

## 5. Result Evaluation

### 5.1. Simulation Setup

The simulation was conducted with a PC using an i7 Processor, 3.1 GHZ CPU, 16 GB mem. and 64-bit Windows 7. The relevant assessment of the approach was performed for FT-Block, and the industrial applications were executed.

1. Performance Metrics: The system throughput helps evaluate FT-Block, latency, and the accuracy of authentication considering different SR, NT, NBT, NU (Number of User), and BS.
  - System Throughput: The performance of the entire system can be improved if the performance of transactions is at very high speed.
  - Transaction Latency: For improving the processing capacity of all the communications, the latency of transactions must be decreased.
  - Accuracy Rate of Authentication: Both FAR as well as MDR comprise the rate of error on an average.

Training of Model Time: To decrease the time taken for training the model for enhancing the user authentication rate and the process of TL.

### 5.2. Results

**Figure 5:**
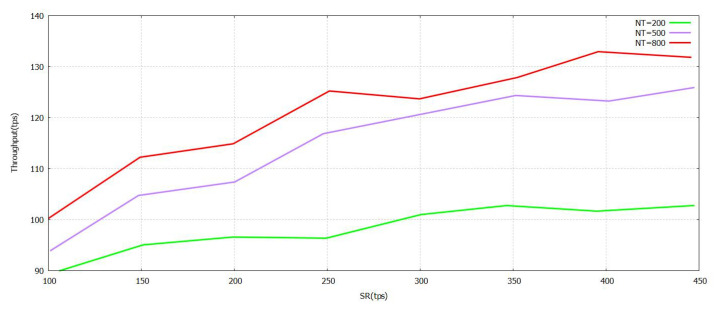
Throughput FT-Block vs. Send Rate with Novel Supportive Twin Delayed DDPG.

**Figure 6:**
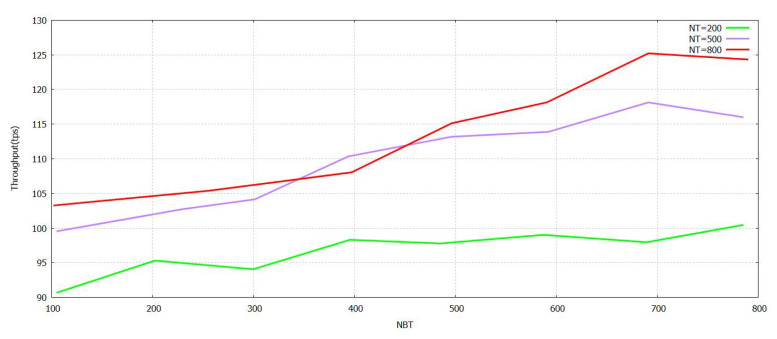
Throughput FT-Block vs. Number of Transactions in Block with Novel Supportive Twin Delayed DDPG.

**Figure 7:**
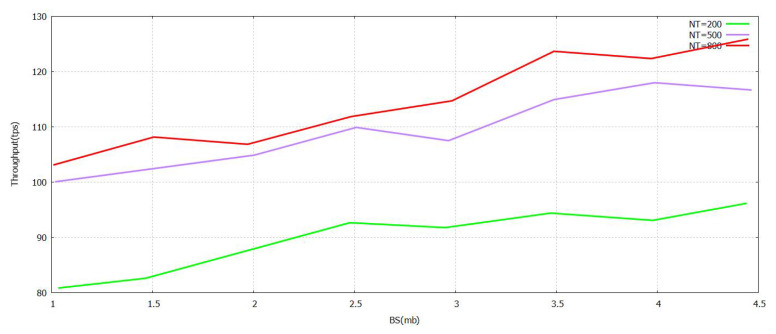
Throughput FT-Block vs. BlockSize with Novel Supportive Twin Delayed DDPG.

The Figure 5 denotes that the progression of the Sending Rate (SR) value grows. The task was performed with the verification in Figure 5 formulated by the proposed FT-Block with trustworthy blockchains. As represented in Figure 6 and Figure 7, either (Block Size) BS or (Number of Block Transaction) NBT increases, thereby increasing the value accordingly with the system throughput for every value of transactions. The value of tps starts to level off when it reaches BS ≥ 3.9 mb and NBT ≥ 700. We attained a very effective user authentication system by presenting TL to trustworthy blockchains; the authentication is efficient as well as reliable for both users of local and foreign contexts. The outcomes represented in Figure 5, Figure 6 and Figure 7 clearly show that the system proposed in FT-Block can definitely give better throughput for all types of industrial applications. This takes place as TL is introduced to implement blockchains that are trustworthy. It enhances the efficiency, authentication, and reliability for local as well as foreign users. The results shown in Figure 5 indicate that FT-Block helps improve the throughputs of the system by facilitating various types of industrial applications. [11]

**Figure 8:**
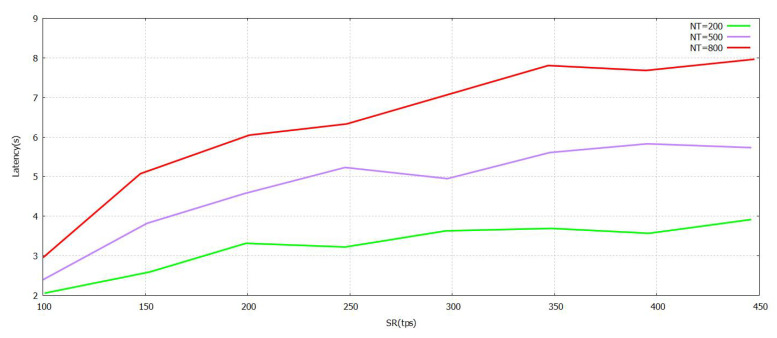
Latency FT-Block vs. Send Rate with Novel Supportive Twin Delayed DDPG.

**Figure 9:**
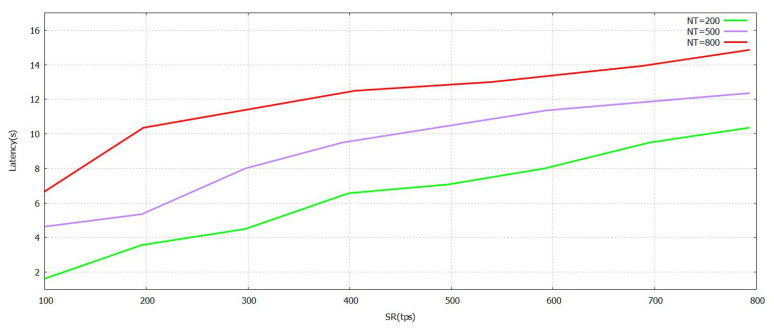
Latency FT-Block vs. Number of Transactions in Block with Novel Supportive Twin Delayed DDPG.

**Figure 10:**
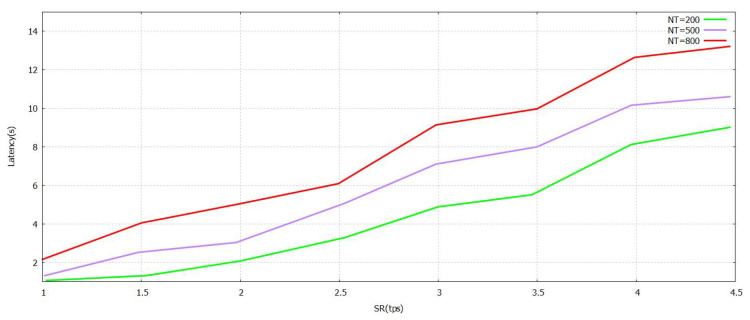
Latency FT-Block vs. BlockSize with Novel Supportive Twin Delayed DDPG.

The latency values are presented with the help of Figure 8, Figure 9 and Figure 10. It has different values of NBT, BS, and SR. A lower level of latency indicates that the performance is enhanced. At first, the block size is set at 2.9 mb. As shown in Figure 8, the transmit rate rises and the delay increases at a predictable pace. When the NT is set to 800, the maximum amount of delay is no more than 8 s. A significant reason for this is that the FT-Block mechanism can help users authenticate and utilize TL efficiently. Figure 8, Figure 9 and Figure 10 show that the blockchain of FT-Block is intelligent and has an excellent capacity to process transactions taking into account several scenarios of industry [11].

**Figure 11:**
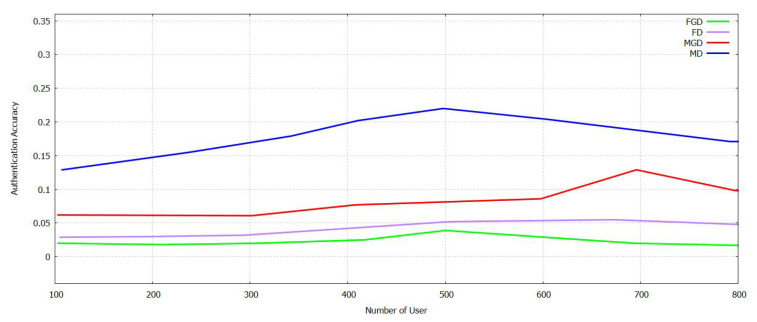
Authentication Accuracy in FAR and MDR with varying Num User with Novel Supportive Twin Delayed DDPG.

**Figure 12:**
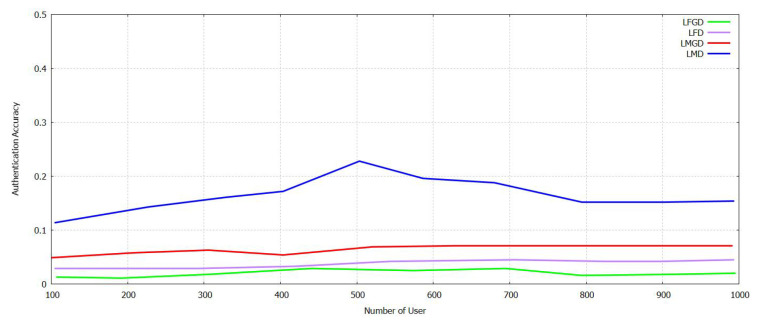
Authentication Accuracy in L_FAR, L_MDR with varying Num User with Novel Supportive Twin Delayed DDPG.

**Figure 13:**
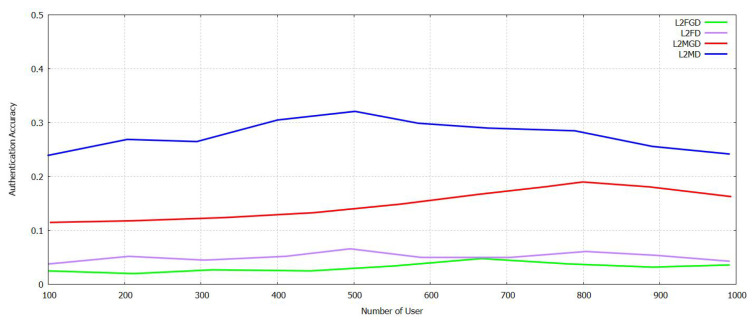
Authentication Accuracy in L2_F and L2_M with varying Num of User with Novel Supportive Twin Delayed DDPG.

The Figure 11 and Figure 12 show a comparison of accuracy calculated between Supportive Twin Delayed DDPG (S-TD3) and DDPG on user authentication for industrial IoTs for False Acceptance Rate (FAR), and Managed Detection and Response (MDR). For L_FAR and L_MDR, when federated transfer learning is deployed locally and helps in authenticating regional users and foreigner users. The users are trained in all of these aspects. The training of the model for R${}_{i}$ regarding the authentication of a user belonging to the local group is shifted to authorize users. L2_F and L2_M Fig. 13 then trains the model trained for the user authentication to be transferred cross-regionally and to be authenticated for locally available users [11]. Then, we contemplate the scenario that accepts the transformed model, based on its locally available user authentication model. As shown in Figure 11, when a certain threshold is reached by the NU, there is an increase in the authentication accuracy of both local and foreign users and gradual improvement in the drop in MDR and FAR. Both MDR and FAR are less for DDPG compared with Supportive Twin Delayed DDPG (S-TD3). It acts as a supportive network and helps discover an accurate threshold of credit to improve the authentication accuracy. It can be shown in Figure 12 that Supportive Twin Delayed DDPG (S-TD3) is affected by the NU, and it can be compared with LCR FAR and MDR that fluctuate in a decreased manner. The Supportive Twin Delayed DDPG (S-TD3) can help achieve an LCR FAR that is less than three percent and an MDR that is less than five percent. This happens as the latter retrains, and the cross-regional model can increase the rate of authentication and its accuracy. In Figure 11 and Figure 12, it is illustrated that FT-Block helps enhance the results and its accuracy in several industrial scenarios [11].

**Figure 14:**
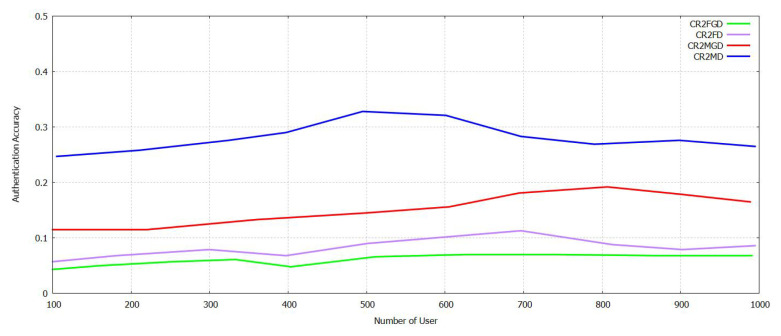
Authentication Accuracy in CR2_F and R2_M with varying Num User with with Novel Supportive Twin Delayed DDPG.

In Figure 13, it can be analyzed that L2_F and L2_M increase at first in the case of NU and then decreases, and then, both are at the same level. Increased levels of authentication and its accuracy require a more significant number of participants. The FAR and MDR are the less compared with DDPG. User authentication based on TL can decrease the time taken for training required by the model in transforming data both locally and across regions. The authentication accuracy depends on the dataset of the user for each of the regions. The Supportive Twin Delayed DDPG (S-TD3) shows the best results compared with shown in Figure 14 DDPG in less CR2_F and CR2_M, i.e., the high CR2_F and CR2_M of the proposed method Supportive Twin Delayed DDPG (S-TD3) algorithm [3,4] are 7.9% and 10.8% comparatively with the existing method DDPG [11]. Evidence to prove the efficiency of the proposed FT-Block for user authentication in industrial IoT is presented.

We witnessed from Table 1 that, with respect to the region number varying all of the metrics correspond with the accuracy of authentication rises.

**Figure 15:**
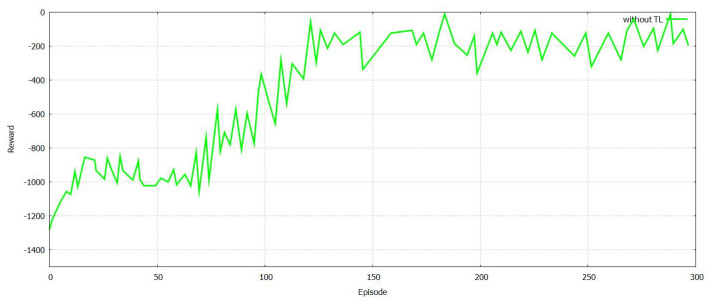
The training time of the model with authentication without federated transfer learning for varied region number.

**Figure 16:**
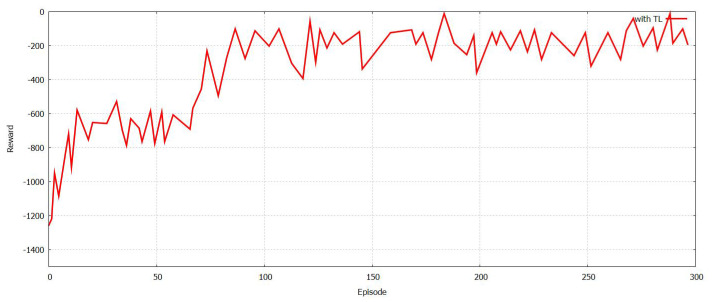
The training time of the model with authentication with transfer learning for varied region number.

The training time was compared and then analyzed with respect to the number of regions with and without TL as shown in Figure 15. It is important to state that a larger NR leads to an increased training model and reduces the training time as well. It is aided by TL as shown in Figure 16, and FT-Block is helpful in authenticating users for several applications of the Internet of things. Additionally, TL can reduce the time for model training that is involved in authentication. An authentication mechanism protects against any spoofing attacks by the [48,49,50,51,52,53] attackers and facilitates physical authentication. The authentication models facilitate reinforced learning, and TL helps in several discoveries. The framework for authentication utilizes specific information related to several devices and prevents all types of physical attacks.

## 6. Conclusions and Future Work

Securing data and maintaining privacy are essential factors in the Internet of things applications and act as foundations for guaranteeing national and industrial security. All industrial applications in the Internet of Things require reliable and real-time information that helps in user interaction and becomes vulnerable to several types of illegal attacks, information leakage, and denying services. It has a security mechanism that is less complex and provides a mechanism of user authentication to solve several security-related problems. A Novel Supportive Twin Delayed DDPG authentication mechanism has been formulated that overcomes the limitations of IIoT-related user mechanisms. This is based on federated transfer learning and proposed by FT-Block. In FT-Block, several blockchains are used efficiently to achieve privacy in application within industries. In FT-Block, several blockchains have been applied to preserve privacy and security for all types of industrial applications. Additionally, by introducing the authentication mechanism based on federated transfer learning, blockchains that are trustworthy to enhance industrial applications’ preservation and security standards are created. Specifically, a deep, supportive gradient-based algorithm trains the user authentication model for specific regions. The proposed Novel Supportive Twin Delayed DDPG, FT-Block facilitates IIoT applications and helps achieve high throughput and low latency. This results in increased accuracy of all authentication metrics as well. The reason behind this is that it brings a greater number of participating users and reduces the difficulties faced by authenticating users. The results that are shown in the Results section indicate an enhanced scale of authentication to the users. Industrial applications such as automated programs such as electrical vehicle can also be deployed with this authentication methodology and can extend the research with vehicular networks, and with transfer learning mechanism, since it provides highest security tradeoff with throughput and latency, the best approach can be fine-tuned for all dynamic networks as future work.

## Figures and Tables

**Table 1:** Accuracy of Authetication vs. Num Reg (NR) with Novel Supportive Twin Delayed DDPG.

Num Reg	5	10	15
LCR_ F/LCR_M	4.2%/5.1%	5.23%/6.15%	5.2%/8.1%
L2_F/L2 MDR	5.2%/6.1%	7.3%/9.1%	8.1%/10.2%
CR2_F/CR2_M	7.15%/8.12%	7.9%/10.8%	8.93%/12.89%

## Data Availability

Not Applicable.
